# Insufficient sleep is prevalent among migraineurs: a population-based study

**DOI:** 10.1186/s10194-017-0756-8

**Published:** 2017-04-28

**Authors:** Jiyoung Kim, Soo-Jin Cho, Won-Joo Kim, Kwang Ik Yang, Chang-Ho Yun, Min Kyung Chu

**Affiliations:** 1 Department of Neurology, Bio Medical Research Institute, Pusan National University Hospital, Pusan National University School of Medicine, Busan, South Korea; 2 Department of Neurology, Dongtan Sacred Heart Hospital, Hallym University College of Medicine, Hwaseong, South Korea; 3 Department of Neurology, Gangnam Severance Hospital, Yonsei University College of Medicine, Seoul, South Korea; 4 Sleep Disorders Center, Department of Neurology, Soonchunhyang University College of Medicine, Cheonan Hospital, Cheonan, South Korea; 5 Department of Neurology, Bundang Clinical Neuroscience Institute, Seoul National University Bundang Hospital, Seongnam, South Korea; 6 Department of Neurology, Kangnam Sacred Heart Hospital, Hallym University College of Medicine, Seoul, South Korea

**Keywords:** Migraine, Sleep, Sleep deprivation, Sleep time, Epidemiology

## Abstract

**Background:**

Sleep disorder and sleep complaints are common in subjects with migraine. Although the association between sleep disorders and migraine has been reported, the association between perceived insufficient sleep and migraine has rarely reported. The aim of this study is to evaluate the association between insufficient sleep and migraine using the data of the Korean Headache-Sleep Study (KHSS).

**Methods:**

The KHSS is a nation-wide cross-sectional population-based survey regarding headache and sleep for Korean adults aged 19 to 69 years. A difference of one hour or more between sleep need and average sleep time indicated insufficient sleep.

**Results:**

Of 2,695 participants, 727 (27.0%) individuals were classified as having insufficient sleep. The prevalence of insufficient sleep among individuals with migraine (45.5%) was significantly higher compared to that among individuals with non-migraine headache (32.9%, *p* = 0.004) or among non-headache (20.4%, *p* < 0.001). Average sleep time did not differ among migraine, non-migraine headache, and non-headache groups (7.3 ± 1.2 vs. 7.2 ± 1.2 vs. 7.3 ± 1.4, *p* = 0.207). Multivariable logistic regression analyses demonstrated that migraine had an increased odds ratio (OR) for insufficient sleep after adjusting for sociodemographic variables, short sleep time, insomnia, poor sleep quality, anxiety, and depression (OR = 1.8, 95% confidence interval [CI] = 1. 2 – 2.7, *p = 0.002*).

**Conclusions:**

The prevalence of insufficient sleep was significantly higher among migraineurs compared to that in non-migraine headache or non-headache group.

## Background

Migraine and sleep disturbance are common complaints in the general population. People who suffer from migraine and sleep disturbance often experience disability and decreased quality of life. Therefore, both conditions impose significant amounts of personal and social burden.

Epidemiological and clinic-based studies have demonstrated a close association between migraine and sleep disturbance. They usually occur in the same individuals [1, 2]. Migraine and insomnia have bidirectional comorbidity, suggesting that they share common mechanisms [3]. Lack of sleep and excessive sleep often trigger migraine attacks [4, 5]. Snoring is a risk factor for transformation from episodic migraine to chronic migraine [6]. Migraineurs report poor sleep quality and daytime tiredness more often than non-migraineurs [7, 8]. Moreover, non-headache symptoms such as tiredness, weary, and yawning have been frequently reported in migraineurs before the headache attack [9, 10]. These symptoms are associated with insufficient sleep.

Adequate or sufficient sleep has been considered as an essential part of optimal health. Individuals with insufficient sleep have higher risk of adverse health outcomes such as hypertension, heart disease, diabetes, depression, obesity, industrial accidents, and occupational errors [11–13]. However, insufficient sleep is a common problem in the general population [14, 15]. It is usually associated with sleep disturbance such as insomnia, excessive daytime sleepiness, and late chronotype [14].

Currently, information on the association between migraine and insufficient sleep is limited. The Korean Headache-Sleep Study (KHSS) is a nation-wide, cross-sectional study regarding headache and sleep. It provides an opportunity to assess the association between insufficient sleep and migraine. The objectives of the present study were: 1) to determine the prevalence of insufficient sleep and migraine in a general population-based sample; 2) to examine the average sleep time and sleep need among individuals with migraine, non-migraine headache, and non-headache; and 3) to assess the association between insufficient sleep and migraine using the data of KHSS.

## Methods

### Study population and survey process

The KHSS is a nation-wide, cross-sectional survey regarding headache and sleep among Korean adults aged 19 to 69 years. It also included items regarding symptoms of anxiety and depression. The study design, methods, and process were described in details previously [16]. Briefly, we used a 2-stage clustered random sampling method for all Korean territories except Jeju-do. It sampled participants proportionally by population distribution. The survey was conducted by door-to-door visit and face-to-face interview by interviewers using a questionnaire. All interviewers were employees of Gallup Korea. They had previous experience of social survey. Data collection of the KHSS was performed from November 2011 to January 2012. The KHSS was approved by the Institutional Review Board and ethics committee of Hallym University Sacred Heart Hospital (IRB No. 2011-I077). Written informed consent was obtained from all participants.

### Migraine assessment

Diagnosis of migraine was based on criteria A to D for migraine without aura (code 1.1) (A, 5 or more attacks in a lifetime; B, attack duration of 4 – 72 h; C, any 2 of the 4 typical headache characteristics [i.e., unilateral pain, pulsating quality, moderate-to-severe pain intensity, and aggravation by routine physical activity]; and D, attacks associated with at least one of the following: nausea, vomiting, or both photophobia and phonophobia) in the second edition of the international classification of headache disorders (ICHD-2) [17]. If a participant reported an experience of headache during the previous year and her/his most severe type headache met all the criteria, she/he was classified as having migraine. We did not investigate the presence of aura because it was difficult to document it in epidemiological study using a questionnaire [18]. Accordingly, participants who were classified as having migraine might have either migraine with aura (code 1.2) or migraine without aura (code 1.1) in the present study. Our survey method has been reported to have a sensitivity of 75.0% and a specificity of 88.2% [19].

### Non-migraine headache assessment

If a participant who was not diagnosed as having migraine responded positively to the question of ‘In the past year, have you had at least 1 headache lasting more than 1 min?’, she or he was classified as having non-migraine headaches.

### Average sleep time and short sleep time assessment

We investigated usual sleep time on workdays and free days. Average sleep time was calculated as (weekday sleep time × 5 + weekend sleep time × 2)/7. If a participant’s average sleep time was ≤ 6 h in a day, she/he was classified as having short sleep time.

### Sleep need and insufficient sleep assessment

We used question “How long do you want to sleep in a day?” to assess sleep need. A difference of one hour or more between sleep need and average sleep time was used to indicate insufficient sleep [14].

### Insomnia and poor sleep quality assessment

We used the Insomnia Severity Index (ISI) to investigate insomnia symptom. ISI is a brief screening questionnaire to measure of insomnia severity. If a participant’s ISI score was 10 or more, she or he was classified as having insomnia [20].

The Pittsburgh Sleep Quality Index (PSQI) was used for assessing poor sleep quality. If an individual’s PSQI score was 6 or more, she/he was classified as having poor sleep quality.

### Anxiety and depression assessment

We used the Goldberg Anxiety Scale (GAS) to investigate anxiety among participants. This comprises four screening items and five supplementary items [21]. Individuals who gave positive answers to two or more screening items and five or more of all scale items were diagnosed with anxiety. The Korean version of the scale has a sensitivity of 82.0% and a specificity of 94.4% for diagnosing anxiety [22]. It has good correlations with the State-Trait Anxiety Inventory, a highly valid tool for assessing anxiety [23].

The Patient Health Questionnaire-9 (PHQ-9) was used to diagnose depression in the present study [24]. Participants who had scores of 10 or more on this measure were considered as having depression. The Korean PHQ-9 has a sensitivity of 81.1% and a specificity of 89.9% for diagnosing depression [25].

### Analyses

Kolmogorov–Smirnov test was used to evaluate the normality of the distribution. After normality was confirmed, Student’s *t*-test or Analysis of Variance was used to compare continuous variables. Post hoc analyses were performed using Turkey’s method. Categorical variables were compared using Chi-square test. Significance level was set at *p < 0.05* for all analyses. Statistical analyses were performed using the Statistical Package for Social Sciences 22.0 (SPSS 22.0; IBM, Armonk, NY, USA).

We evaluated odds ratios (ORs) with 95% confidence interval (CI) for the occurrence of insufficient sleep through univariable and multivariable logistic regression analyses. In univariable analyses, we modelled the ORs for sleep insufficiency without adjusting for covariates. In multivariable analyses, we used 4 models. In Model 1, adjustment was conducted for sociodemographic variables (age, gender, size of residential area, educational level and monthly income level). Model 2 incorporated short sleep time (≤6 h in average sleep time), insomnia, and poor sleep quality (PSQI) to Model 1. Model 3 included anxiety (GAS) and depression (PHQ-9) to Model 1. The final model, Model 4, incorporated sociodemographic variables, short sleep time, insomnia, poor sleep quality, anxiety, and depression.

## Results

### Survey

**Fig. 1:**
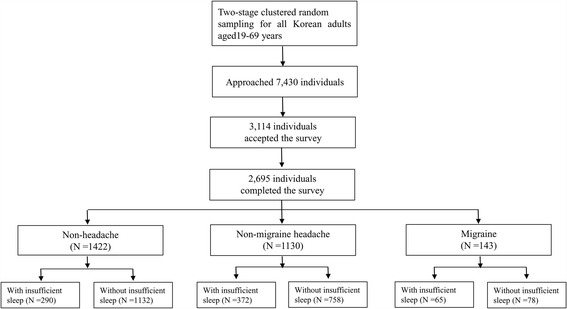
Flow chart depicting participation in the present study

Interviewers approached a total of 7,430 individuals. Of them, 2,695 subjects completed the survey (cooperation rate: 36.3%, Fig. 1). The distribution of age, gender, size of residential area, educational level or monthly income level of our sample was not significantly different from that of the general population of Korea (Table 1).

**Table 1:** Sociodemographic characteristics of survey participants, total Korean population, and cases identified as having migraine, non-migraine headache, or insufficient sleep

	Survey participants *N* (%)	Total population *N* (%)	*P*	Migraine *N*, % (95% CI)	Non-migraine headache *N*, % (95% CI)	Insufficient sleep *N*, % (95% CI)
Gender						
Men Women	1,345 (49.3)	17,584,365 (50.6)	0.854^a^	36, 2.7 (1.8–3.5)	471, 35.0 (32.4–37.6)	329, 24.5 (22.2–26.8)
	1,350 (50.7)	17,198,350 (49.4)		107, 7.9 (6.5–9.4)	659, 49.0 (46.2–51.5)	398, 29.5 (27.0–31.9)
Age						
19–29	542 (20.5)	7,717,947 (22.2)	0.917^a^	25, 4.5 (2.7–6.2)	231, 42.6 (38.4–46.8)	147, 27.1 (23.4–30.9)
30–39	604 (21.9)	8,349,487 (24.0)		42, 7.0 (4.9–9.1)	269, 44.5 (40.6–48.5)	174, 28.8 (25.1–32.4)
40–49	611 (23.1)	8,613,110 (24.8)		39, 6.5 (4.5–8.4)	277, 45.3 (41.4–49.3)	186, 30.4 (26.7–34.1)
50–59	529 (18.9)	6,167,505 (17.7)		22, 4.1 (2.4–5.9)	204, 38.6 (34.4–42.7)	140, 26.5 (22.7–30.2)
60–69	409 (15.6)	3,934,666 (11.3)		15, 3.9 (2.0–5.7)	149, 36.4 (31.7–41.1)	80, 20.0 (15.7–23.4)
Size of residential area						
Large city	1,248 (46.3)	16,776,771 (48.2)	0.921^a^	76, 6.1 (4.8–7.5)	525, 42.1 (39.3–44.8)	334, 26.8 (24.3–29.2)
Medium-to-small city	1186 (44.0)	15,164,345 (43.6)		48, 4.0 (2.9–5.2)	488, 41.1 (38.3–43.9)	327, 27.6 (25.0–30.1)
Rural area	261 (9.7)	2,841,599 (8.2)		19, 7.4 (4.2–10.6)	117, 44.8 (38.8–50.9)	66, 25.3 (20.0–30.6)
Education level						
Middle school or less	393 (14.9)	6,608,716 (19.0)	0.752^a^	22, 5.5 (4.2–7.7)	165, 42.0 (37.1–46.9)	90, 22.9 (18.7–27.1)
High school	1,208 (44.5)	15,234,829 (43.8)		60, 5.0 (3.8–6.3)	502, 41.6 (38.8–44.3)	345, 28.6 (26.0–31.1)
College or more	1,068 (39.6)	12,939,170 (37.2)		60, 5.6 (4.3–7.0)	457, 42.7 (39.8–45.8)	286, 26.7 (24.1–29.4)
Not responded	26 (1.0)			1, 3.8 (0.0–11.8)	6, 5.3 (0.3–9.6)	6, 23.1 (5.7–40.4)
Monthly income (Korean Won^b^)						
< 2,000,000	403(14.8)	4,452,188 (12.8)	0.503^a^	21, 14.7 (9.1–20.3)	169, 15.0 (13.1–17.1)	86,11.8 (9.6–14.3)
2,000,000–4,999,999	1,813(67.3)	24,417,466 (70.2)		96, 67.1 (59.4–74.8)	751, 66.5 (63.8–69.1)	499, 68.6 (65.2–71.9)
≥ 5000000	422(15.7)	5,913,062 (17.0)		22, 15.4 (9.8–21.7)	186, 16.5 (14.3–18.7)	124, 17.1 (14.3–19.9)
Not responded	57(2.1)			4, 2.8 (0.7–5.6)	24, 2.1 (1.3–3.0)	18, 2.5 (1.4–3.7)
Total	2695 (100.0)	34,782,715 (100.0)		143, 5.3 (4.5–6.2)	1130, 41.9 (40.0–43.8)	727, 27.0 (25.3–28.7)

*CI* Confidence Interval

^a^Comparison of gender, age group, size of residential area, educational level and monthly income level distributions between the sample in the present study and the total population of Korea

^b^1USD = 1,123 Korean Won (February 1, 2012)

### Prevalence of migraine, non-migraine headache, and insufficient sleep

Of the 2,695 participants, 143 (5.3%) subjects were classified as having migraine while 1,130 (41.9%) individuals and 1,422 (52.8%) individuals were classified as non-migraine headache and non-headache, respectively. Seven hundred and twenty-seven (27.0%) individuals answered that they needed more than 1 h of sleep compared to their average sleep time. Therefore, they were classified as having insufficient sleep (Table 1).

### Average sleep time, short sleep time, and sleep need

The average sleep time of all participants was 7.3 ± 1.2 h in a day. The average sleep time was not significantly different among individuals with migraine, those with non-migraine headache, and non-headache (7.3 ± 1.4 vs. 7.2 ± 1.2 vs. 7.3 ± 1.2 h, *p = 0.207*). Four hundred and sixty-nine (17.4%, 95% CI = 16.0% - 18.8%) individuals were classified as having short sleep time. The prevalence of short sleep time was not significantly different among individuals with migraine, those with non-migraine headache, and the non-headache (18.2% vs. 19.1% vs. 16.0%, *p = 0.110*).

Sleep need of migraineurs (8.4 ± 1.5 h) was significantly longer compared to sleep need of those with non-migraine (8.0 ± 1.3 h, *p < 0.001*) and that of non-headache individuals (7.8 ± 1.3 h, *p < 0.001*). Among individuals with non-migraine headache, sleep need was longer (*p < 0.001*) than that of individuals with non-headache (Table 2).

**Table 2:** Average sleep time and sleep need of individuals with migraine, non-migraine, or non-headache

	Non-headache (1)	Non-migraine headache (2)	Migraine (3)	*P-value*	*Post-hoc* analysis
Average sleep time, hours	7.3 ± 1.2	7.2 ± 1.2	7.3 ± 1.4	*0.207*	
Sleep need, hours	7.8 ± 1.3	8.0 ± 1.3	8.4 ± 1.5	*<0.001*	(1) vs. (2) <*0.001*
					(1) vs. (3) <*0.001*
					(2) vs. (3) <*0.001*

Values are expressed as means ± SD. *SD* Standard Deviation

### Insomnia and poor sleep quality

Among 2,695 participants, 290 (10.8%, 95% CI = 9.6% – 11.9%) and 715 (26.5%, 95% CI = 24.8% – 28.2%) individuals were classified as having insomnia and poor sleep quality, respectively. Insomnia was more prevalent in individuals with migraine (25.9%, 95% CI = 18.9% – 32.9%, *p < 0.001*) or with non-migraine headache (15.1%, 95% CI = 12.8% – 17.5%, *p < 0.001*) compared to that in non-headache (5.8%, 95% CI = 4.6% – 6.9%). Poor sleep quality was more prevalent in individuals with migraine (47.6%, 95% CI = 39.3% – 55.8%, *p < 0.001*) or with non-migraine headache (30.9%, 95% CI = 28.2% – 33.6%, *p* < 0.001) compared to that in individuals with non-headache (21.0%, 95% CI = 18.8% – 23.1%). Moreover, the prevalence of poor sleep quality in individuals with migraine was significantly higher compared to that in individuals with non-migraine headache (*p = 0.037*).

### Insufficient sleep and migraine

**Fig. 2:**
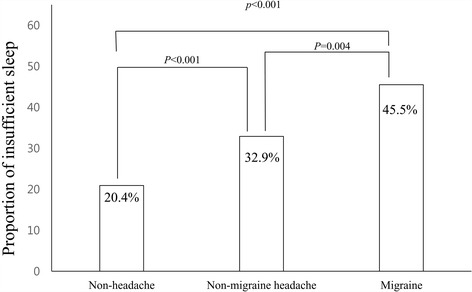
Prevalence of insufficient sleep among participants with migraine, those with non-migraine headache and non-headache

Among the 143 migraineurs, 65 (45.5%, 95% CI = 37.2% – 53.7%) individuals were classified as having insufficient sleep. Among the 1,130 individuals with non-migraine headache, 372 (32.9%, 95% CI = 30.2% – 35.7%) had insufficient sleep. Among the 1,422 individuals with non-headache, 290 (20.4%, 95% CI =18.3% – 22.5%) individuals were found to have insufficient sleep. The prevalence of insufficient sleep was significantly (*p = 0.004*) higher among individuals with migraine compared to that in individuals with non-migraine headache. Additionally, individuals with non-migraine headache had higher (*p* < *0.001*) prevalence of insufficient sleep compared to those with non-headache (Fig. 2).

Among individuals with insufficient sleep, average sleep time was not significantly different among individuals with migraine, those with non-migraine headache, and non-headache (6.6 ± 1.2 vs. 6.5 ± 1.3 vs. 6.6 ± 1.1 h, *p = 0.875*). Among individuals without insufficient sleep, average sleep time of migraineurs was longer than those with non-headache (7.9 ± 1.2 vs. 7.6 ± 1.1 h, *p = 0.029*). However, average sleep time of individuals with migraine did not significantly differ from that of individuals with non-migraine headache (7.9 ± 1.2 vs. 7.5 ± 1.2 h, *p = 0.109*).

Prevalence of insufficient sleep was analyzed according to headache frequency and headache intensity among migraineurs. Prevalence of insufficient sleep in participants with migraineurss with 1–14 attacks per month (44.8%, *p = 0.549*) and participants with migraine with ≥15 attacks per month (55.6%, *p* = 0.987) was not significantly different compared to that of those with <1 attack per month (44.7%). Prevalence of insufficient sleep was not significantly different among migraineurs according to mild, moderate and severe headache intensity (46.4% vs. 45.3% vs. 44.8%, *p = 0.992*).

### Univariable and multivariable analyses for insufficient sleep

In univariable analyses, migraine showed an increased OR for insufficient sleep (OR = 2.4, 95% CI = 1.7 – 3.3, *p < 0.001*). In addition, short sleep time (OR = 5.2, 95% CI = 4.2 – 6.4, *p* < *0.001*), insomnia (OR = 3.9, 95% CI = 3.0 – 5.0, *p < 0.001*), poor sleep quality (OR = 3.2, 95% CI = 2.7 – 3.9, *p* < *0.001*), anxiety (OR = 2.7, 95% CI = 2.0 – 3.4, *p* < *0.001*), and depression (OR = 2.7, 95% CI = 1.8 – 3.9, *p < 0.001*) showed increased ORs for insufficient sleep. In multivariable analyses after adjusting for sociodemographic variables (age, gender, size of residential area, educational level and monthly income level; Model 1), migraine showed an increased OR for insufficient sleep (OR = 2.3, 95% CI = 1.6 –3.1, *p < 0.001*). In Model 2 after adjusting for sociodemographic variables, poor sleep quality, short sleep time, insomnia, and poor sleep quality (OR = 1.9, 95% CI = 1.3 – 2.8, *p = 0.001*) and Model 3 after adjusting for sociodemographic variables, anxiety, and depression (OR = 1.8, 95% CI = 1.3 – 2.6, *p = 0.001*), migraine maintained increased ORs for insufficient sleep. After adjusting for sociodemographic variables, short sleep time, insomnia, poor sleep quality, anxiety, and depression (Model 4), migraine was still a significant factor for insufficient sleep (OR = 1.8, 95% CI = 1.2 – 2.7, *p = 0.002*, Table 3).

**Table 3:** Univariable and multivariable analyses for insufficient sleep after adjusting for sociodemographics, short sleep time, insomnia, poor sleep quality, anxiety, and depression

	Univariable analysis OR (95%CI)	Multivariable analysis			
		Model 1 OR (95%CI)	Model 2 OR (95%CI)	Model 3 OR (95%CI)	Model 4 OR (95%CI)
Migraine	2.4 (1.7–3.3), *p < 0.001*	2.3 (1.6–3.3), *p < 0.001*	1.9 (1.3–2.8), *p = 0.001*	1.8 (1.3–2.6), *p = 0.001*	1.8 (1.2–2.7), *p = 0.002*
Short sleep time	5.2 (4.2–6.4), *p < 0.001*		4.5 (3.5–5.7), *p < 0.001*		4.5 (3.6–5.8), *p < 0.001*
Insomnia	3.9 (3.0–5.0), *P < 0.001*		2.6 (1.9–3.5), *P < 0.001*		2.4(1.8–3.4), *p < 0.001*
Poor sleep quality	3.2 (2.7–3.9), *p < 0.001*		1.6 (1.3–2.1), *p < 0.001*		1.6 (1.3–2.0) *p < 0.001*
Anxiety	2.7 (2.0–3.4), *p < 0.001*			2.4 (1.8–3.1), *p < 0.001*	1.6 (1.2–2.2) *p = 0.004*
Depression	2.7 (1.8–3.9), *p < 0.001*			1.6(1.1–2.5), *p = 0.022*	0.8(0.5–1.3), *p = 0.321*

Model 1 adjusted for sociodemographics (age, gender, size of residential area, education level and monthly income level)

Model 2 adjusted for sociodemographics, short sleep time, insomnia and poor sleep quality

Model 3 adjusted for sociodemographics, anxiety, and depression

Model 4 adjusted for sociodemographics, short sleep time, insomnia,poor sleep quality, anxiety, and depression

*OR* Odds Ratio, *CI* Confidence Interval

## Discussion

The key findings of this study were: (1) The prevalence of insufficient sleep and migraine were 27.0% and 5.3%, respectively; (2) Although average sleep time was not significantly different according to headache type, sleep need of migraineurs was longer than that of individuals with non-migraine headache or non-headache; (3) The prevalence of insufficient sleep was significantly higher in migraineurs (45.5%) compared to that in those with non-migraine headache (32.9%) or non-headache (20.4%).

This study demonstrated that migraineurs more frequently had insufficient sleep than individuals with non-migraine headache or non-headache. Enough sleep time and good sleep quality are essential for sufficient sleep [13, 26]. Sleep time did not differ according to headache type in the present study. Therefore, poor sleep quality with similar sleep time among migraineurs might have resulted in insufficient sleep. We also observed frequent poor sleep quality among migraineurs in the present study.

In multivariable logistic regression analyses for insufficient sleep in our study, migraine increased the OR for insufficient sleep even after adjusting for sociodemographic variables, short sleep time, insomnia, poor sleep quality, anxiety, and depression. This finding might be contradictory to our assumption that insufficient sleep among migraineurs resulted from poor sleep quality. One possible explanation for the discrepancy was that insufficient sleep among migraineurs was not only due to poor sleep quality, but also due to the fact that migraine itself was associated with insufficient sleep. Another possible explanation might be due to sleep quality assessment instrument. We used PSQI to assessing sleep quality in the present study. Although PSQI is a widely using instrument for assessing sleep quality, PSQI might not properly reflect sleep quality of migraineurs. Further studies regarding sleep quality among migraineurs using other validated sleep quality assessing instruments are needed.

In the present study, migraineurs had more sleep need compared to individuals with non-migraine headache and non-headache. One possible explanation for increased sleep need among migraineurs is migraineurs’ attempt to sleep for relieving migraine attacks. Migraineurs often report relieving migraine symptoms by sleep [27, 28]. Therefore, migraineurs may need longer sleep time than non-migraineurs for relieving their migraine attacks. Another possible explanation for increased sleep need among migraineurs is their decreased sleep quality. Decreased sleep quality among migraineurs has been repeatedly reported [7, 8]. Accordingly, migraineurs need more sleep for sufficient sleep.

The demonstration of insufficient sleep among migraineurs in the present study was compatible with previous electrophysiological studies regarding the relationship between sleep and migraine [29, 30]. A case-controlled study including 53 migraineurs and 34 controls has reported that migraineurs have higher awaking index, strong tendency for more slow wave sleep, and lower fast arousal index during interictal period [29]. In addition, migraineurs have less sleep latency compared to controls during the preictal period. These findings have been interpreted as migraineurs commonly suffer from relative sleep deprivation and need more sleep than healthy controls [29]. Another case-controlled study has revealed that non-sleep-related migraine patients have more slow wave sleep and higher K-burst index compared to sleep-related migraine patients or controls, indicating more sleep deprivation [30].

The overall prevalence of insufficient sleep was 27.0% in present study. This prevalence was similar to that reported in previous studies [14, 31, 32]. A survey in Finland has revealed that the prevalence of insufficient sleep is 20.4% among those aged 33 to 60 years [14]. A telephone survey in Australia has shown that insufficient sleep prevalence is 28% [31]. A Swedish population survey for people aged 30 to 65 years has revealed that 28% of females and 21% of males have experienced too little sleep [32].

In present study, the 1-year prevalence of migraine (5.3%) is lower than that of European (10–25%) and North American (9–16%) studies [33]. The 1-year prevalence of migraine in Asian countries has been found to range between 4.7% and 9.1% in most studies [19, 34]. Therefore, the 1-year prevalence of migraine in the present study was similar to that in Asian countries. Similarity in the prevalence of insufficient sleep and migraine between our study and previous studies suggested that insufficient sleep and migraine were properly evaluated in the present study.

The overall response rate in the present study was not high. However, we used two-stage clustered random sampling, proportional to the population distribution of Korea. Therefore, distribution of age, sex, size of residential area, educational level and monthly income level of our participants was not different from those of Korean general population. In addition, the prevalence of migraine, anxiety, depression, poor sleep quality and insomnia in the data of KHSS were similar to that in previous population-based studies [16, 35, 36] Therefore, we could assure that we successfully investigated migraine and insufficient sleep in the present study.

This study has some limitations. First, sleep parameters such as total sleep time and sleep quality were not evaluated by objective methods such as polysomnography (PSG). We evaluated sleep habits and sleep quality with self-reported questionnaires. In population based study, it is practically difficulty to perform PSG to evaluate sleep parameters and sleep quality in all participants. Second, although the present study was a large population-based study, some subgroup analyses might not have statistical significance due to the limited sample size. In other words, the lack of significant findings in subgroup analyses could be due to limited sample size.

The present study also has some strength. First, the present study had a large sample size. The distribution of age, gender, size of residential area, education level and monthly income level in participants represented the Korean general population. Second, this study evaluated covariates such as sociodemographic variables, short sleep time, insomnia, poor sleep quality, anxiety, and depression which could affect insufficient sleep. The relationship between migraine and insufficient sleep was significantly maintained after adjusting for covariates.

## Conclusion

Approximately half of migraineurs experienced insufficient sleep. The prevalence of insufficient sleep was higher in individuals with migraine compared to that in those with non-migraine headache or non-headache. Although the average sleep time was not significantly different among individuals with migraine, those with non-migraine headache, and non-headache, sleep need was longer among migraineurs than that among individuals with non-migraine or non-headache. Our findings suggest that migraineurs need longer sleep time than individuals with non-migraine or non-headache to have sufficient sleep.

## Abbreviations

CI: Confidence interval
GAS: Goldberg anxiety scale
ICHD: International classification of headache disorders
ISI: Insomnia severity index
KHSS: Korean headache-sleep study
OR: Odds ratio
PHQ-9: Patient health questionnaire-9
PSG: Polysomnography
PSQI: Pittsburgh sleep quality index score
